# Psychological Distress and Weight Gain in Pregnancy: a Population-Based Study

**DOI:** 10.1007/s12529-019-09832-0

**Published:** 2019-12-18

**Authors:** Florianne O. L. Vehmeijer, Sangeeta R. Balkaran, Susana Santos, Romy Gaillard, Janine F. Felix, Manon H. J. Hillegers, Hanan El Marroun, Vincent W. V. Jaddoe

**Affiliations:** 1 The Generation R Study Group, Erasmus MC, University Medical Center, Rotterdam, The Netherlands; 2 Department of Epidemiology, Erasmus MC, University Medical Center, Rotterdam, The Netherlands; 3 Department of Pediatrics, Erasmus MC, University Medical Center, Rotterdam, The Netherlands; 4 Department of Child and Adolescent Psychiatry/Psychology, Erasmus MC, University Medical Center, Rotterdam, The Netherlands

**Keywords:** Psychological distress, Depression, anxiety, Pregnancy, Gestational weight gain

## Abstract

**Background:**

Psychological distress and inappropriate or excessive weight gain are common in pregnancy and are associated with adverse maternal and offspring outcomes. Psychological well-being and weight status of women during pregnancy might be interrelated. We aimed to examine whether psychological distress during pregnancy is associated with gestational weight gain.

**Method:**

In a population-based cohort of 3393 pregnant women, information about psychological distress, depressive and anxiety symptoms was assessed at 20 weeks of gestation using the Brief Symptom Inventory questionnaire. Weight was repeatedly measured during pregnancy and obtained by questionnaire before and after pregnancy. Linear regression and multinomial logistic regression models were used. Weight gain in the second half of pregnancy, total weight gain, and the risks of inadequate and excessive total weight gain were the main outcome measures.

**Results:**

In total, 7.0% of all women experienced psychological distress. Overall psychological distress and anxiety were associated with lower weight gain in the second half of pregnancy (differences − 1.00 kg (95% confidence interval (CI) − 1.62, − 0.37) and − 0.68 kg (95% CI - 1.24, -0.11), respectively). These associations fully attenuated into non-significance after taking account for socio-demographic variables. Similar results were observed for total weight gain. Only women with anxiety symptoms had, independently of potential confounders, a lower risk of excessive weight gain (odds ratio (OR) 0.61 (95% CI 0.48, 0.91)).

**Conclusions:**

In this large prospective cohort study, the observed associations of psychological distress with weight gain during pregnancy seem to be largely explained by common socio-demographic factors.

**Electronic supplementary material:**

The online version of this article (10.1007/s12529-019-09832-0) contains supplementary material, which is available to authorized users.

## Introduction

Psychological distress is generally defined as general stress, depressive symptoms, anxiety or experiencing an adverse life event [1, 2]. In western countries, 5–20% of pregnant women experience psychological distress [2–4]. Also, more than 60% of pregnant women have either inadequate or excessive weight gain [5, 6]. Pregnancy is a critical period for psychological distress and weight gain, since both are associated with increased risks of adverse maternal and offspring outcomes [7–11]. Previously, we have reported that, compared with low or recommended weight gain, excessive weight gain was associated with a higher risk of gestational hypertension, cesarean delivery, large size for gestational age infants, and childhood overweight [12]. We have also reported that anxiety and depression during pregnancy were associated with impaired fetal growth [13]. Psychological distress and weight gain in pregnant women may also affect each other [14–17]. Two systematic reviews among, in total, 12 studies have been performed on the association between psychological distress and weight gain in pregnancy. One systematic review showed no association and the second systematic review only reported an association of depression, but not psychological distress and anxiety, with increased gestational weight gain [7, 9]. These reviews compiled studies with a modest sample size, used different definitions of psychological distress, depression and anxiety and did not define cutoffs for psychological distress to consider clinical importance.

We hypothesized that psychological distress during pregnancy is associated with gestational weight gain. We examined in a population-based prospective cohort study among 3393 pregnant women the associations of psychological distress during pregnancy and gestational weight gain. We also explored whether any association was explained by socio-demographic or lifestyle-related variables.

## Methods

### Study Design

This study was embedded in the Generation R Study, a population-based prospective cohort study from early pregnancy until young adulthood in Rotterdam, the Netherlands [18, 19]. The study was approved by the Medical Ethics Committee of Erasmus Medical Center Rotterdam, The Netherlands (MEC 198.782/2001/31). Pregnant women were enrolled between 2002 and 2006. Written informed consent was obtained from all women in the study. In total, 8879 mothers (91% of the full cohort) were enrolled during pregnancy of whom information about psychological distress during pregnancy was available in 6650. We further excluded pregnancies not leading to singleton live births (*N* = 101) and women without information on weight gain during pregnancy (*N* = 3156). This resulted in a population for analysis of 3393 mothers.

### Psychological Distress During Pregnancy

The Brief Symptom Inventory (BSI) questionnaire was used to examine psychological distress at approximately 20 weeks of gestation. The BSI is a validated self-report questionnaire consisting of 53 items, describing multidimensional psychopathologic problems and complaints in adults in the preceding 7 days [20–23]. The items are divided in 9 subscales (including anxiety, depression, hostility, phobic anxiety, interpersonal sensitivity, obsessive-compulsiveness, paranoid ideation, psychoticism, somatization). As an indicator of overall psychological distress, we used the Global Severity Index (GSI) that is a total score of the 9 subscales (all 53 items of the BSI). Additionally, we used the depression and anxiety subscales separately. We chose these 2 subscales because they are widely used as valid proxies for psychological distress during pregnancy [1, 2]. The items were rated on a 5-point unidimensional scale ranging from “0” (not at all) to “4” (extremely). A total score was calculated for each symptom scale by summing the item scores of the scales and dividing the results by the number of items in that scale. Higher scores represented an increased occurrence of symptoms. Psychological symptoms were dichotomized (yes/no) by using the following clinical cutoffs derived from a psychiatric outpatient sample of Dutch non-pregnant women: 0.71 for overall psychological symptoms scale, 0.80 for the depression symptoms scale and 0.71 for the anxiety symptoms scale [23].

### Weight Measurements During Pregnancy

As enrolment in our study was in pregnancy, we were not able to measure weight before pregnancy. Information on pre-pregnancy weight was obtained by questionnaires at enrollment. Pre-pregnancy body mass index (BMI) in kg/m^2^ was calculated using pre-pregnancy weight (kg) as reported by the mother and height (m) measured at enrolment and was categorized into underweight (< 18.5 kg/m^2^), normal weight (18.5–24.9 kg/m^2^), overweight (25–29.9 kg/m^2^), and obesity (≥ 30 kg/m^2^) according to the World Health Organization (WHO) 2000 criteria [24]. Weight measurements during pregnancy were performed in a dedicated research center without shoes and heavy clothing. Since we collected information about psychological distress at 20 weeks, we used weight gain in the second half of pregnancy as main outcome. Weight gain in the second half of pregnancy was calculated subtracting weight measured in mid-pregnancy (median 20.4 weeks (95% range 18.6–24.9 weeks)) from the maximum reported weight in pregnancy. The latest weight before delivery was obtained by questionnaire completed 2 months after delivery, hereafter referred to as maximum reported weight in pregnancy. Total weight gain during pregnancy was calculated by subtracting reported pre-pregnancy weight from the maximum reported weight in pregnancy. Total gestational weight gain was categorized into inadequate, adequate, and excessive weight gain following the 2009 Institute of Medicine (IOM) weight gain recommendation criteria using both categorized pre-pregnancy BMI and total gestational weight gain [25–27].

### Covariates

Gestational age was established using the last menstrual period or first trimester ultrasound measurement [28]. We obtained information on maternal age, ethnicity, parity, educational level, and marital status by questionnaire at enrolment [18]. Information about folic acid intake, smoking, alcohol consumption and nutritional intake in kcal (kilocalories) was assessed by questionnaires during pregnancy.

### Statistical Analysis

First, we compared subject characteristics between women with and those without psychological distress using Pearson’s chi-square tests, independent sample *t* tests and Mann-Whitney tests. We performed non-response analysis using the same tests to assess differences between women with and without information on weight gain during pregnancy. We compared psychological distress and weight gain characteristics between pre-pregnancy BMI categories. Second, we used linear regression models to assess the associations of overall psychological distress, depression, and anxiety with weight gain in the second half of pregnancy and total gestational weight gain. We examined potential interactions of maternal psychological distress with pre-pregnancy BMI and ethnicity in the association with gestational weight gain. We observed a statistically significant interaction of maternal psychological distress and pre-pregnancy BMI and thus performed stratified analyses for the clinical BMI categories according to the WHO 2000 criteria (underweight, normal weight, overweight, obesity). Further, we performed sensitivity analyses among full-term mothers, defined as a gestational age of ≥ 37 weeks at birth, to exclude potential bias by preterm birth. Third, we used multinomial logistic regression models to assess the associations of psychological distress, depression, and anxiety with clinical categories of gestational weight gain according to the IOM criteria (inadequate, adequate and excessive weight gain). For all regression models, the basic models were adjusted for maternal age, whereas the full models were adjusted for potential confounders. We included covariates in the models if they were associated with psychological distress and gestational weight gain or if they changed the effect estimates substantially (> 10%). In order to maintain statistical power and reduce bias related to missing data on covariates (missing data on covariates ranged from 0 to 21%, see Electronic Supplementary Material 2), we performed multiple imputation using the Markov Chain Monte Carlo method. Exploratory analyses showed the data was not missing completely at random (MCAR) (indicated by the Little’s test, *p* value < 0.05) [29]. Comparison between characteristics of complete cases (participants with no missing data) and participants with at least one missing value showed no large differences. Considering these results and no likely reason for the data to be missing not at random (MNAR), we proceeded with multiple imputation for which missing at random (MAR) is an assumption [30]. Five new datasets were created and pooled results are presented. No major differences in descriptive statistics were found between the original and imputed datasets. Statistical analyses were performed with the Statistical Package of Social Sciences version 21.0 for Windows (IBM Corp, Armonk, NY, USA).

## Results

### Subject Characteristics

Psychological distress, depression, and anxiety were reported by 7.0%, 7.0%, and 8.4% of all pregnant women, respectively. Table 1 shows the subject characteristics. In total, 20.1% and 45.0% of all women had inadequate and excessive gestational weight gain, respectively. Compared to women without psychological distress, those with psychological distress were more often younger, lower educated, without a partner, of a non-Dutch ethnicity, continued smokers, and had a lower nutritional intake, alcohol, and folic acid supplement use (Table 1). Table 2 shows that the prevalence of psychological distress, depression, and anxiety varies between BMI categories. Women with a normal pre-pregnancy weight had the lowest prevalence of psychological distress and the highest mean weight gain.

**Table 1:** Characteristics of study population (*N* = 3393)^a^

	Population for analysis (*N* = 3393)	Psychological distress (*N* = 238)	No psychological distress (*N* = 3155)	*P*-value^b^
Age at intake, mean (SD), years	31.0 (4.7)	28.2 (5.8)	31.3 (4.5)	< 0.001
Pre-pregnancy weight, median (95% range), kg	64.0 (49.0, 97.0)	62.0 (47.0, 104.6)	64.0 (49.9, 96.2)	< 0.01
Pre-pregnancy BMI, median (95% range) kg/m^2^	22.3 (18.2, 33.6)	22.7 (17.9, 36.7)	22.3 (18.2, 33.3)	< 0.01
Pre-pregnancy BMI clinical categories, *N* (%)				< 0.01
Underweight	111 (3.8)	9 (4.4)	102 (3.8)	
Normal weight	2127 (73.0)	140 (68.6)	1987 (73.3)	
Overweight	491 (16.8)	40 (19.6)	451 (16.6)	
Obesity	185 (6.3)	15 (7.4)	170 (6.3)	
Gestational age at birth, median (95% range), weeks	40.1 (36.3, 42.4)	40.3 (36.1, 42.6)	40.0 (36.3, 42.4)	0.21
Total weight gain, mean (SD), kg	14.9 (5.9)	14.4 (6.7)	15.0 (5.8)	0.17
Gestational weight gain clinical categories (IOM criteria), *N* (%)				0.21
Inadequate weight gain	586 (20.1)	49 (24.0)	537 (19.8)	
Adequate weight gain	1018 (34.9)	74 (36.3)	944 (34.8)	
Excessive weight gain	1310 (45.0)	81 (39.7)	1229 (45.4)	
Weight gain 2nd half of pregnancy, mean(SD), kg	9.6 (4.6)	9.0 (4.8)	9.6 (4.6)	
Parity, *N* (%)				0.23
Nulliparous	2059 (60.7)	144 (60.5)	1915 (60.7)	
Multiparous	1334 (39.7)	94 (39.5)	1240 (39.3)	
Education, *N* (%)				< 0.001
Primary school	178 (5.3)	31 (13.0)	147 (4.7)	
Secondary school	1280 (37.7)	138 (58.0)	1142 (36.2)	
Higher education	1935 (57.0)	69 (29.0)	1866 (59.1)	
Marital status, *N* (%)				< 0.001
Married/living together	3084 (90.9)	185 (77.7)	2899 (91.9)	
No partner	309 (9.1)	53 (22.3)	256 (8.1)	
Ethnicity, *N* (%)				< 0.001
Dutch-European	2448 (72.1)	92 (38.6)	2356 (74.6)	
Surinamese	200 (5.9)	28 (11.8)	172 (5.5)	
Turkish	178 (5.2)	46 (19.3)	132 (4.2)	
Moroccan	109 (3.2)	24 (10.1)	85 (2.7)	
Cape Verdian	70 (2.1)	19 (8.0)	52 (1.6)	
Dutch Antilles	69 (2.1)	6 (2.5)	62 (2.0)	
Others	319 (9.4)	23 (9.7)	296 (9.4)	
Alcohol consumption, *N* (%)				< 0.001
No	1304 (38.4)	130 (54.6)	1173 (37.2)	
Yes	2089 (61.6)	108 (45.4)	1982 (62.8)	
Smoking habits, *N* (%)				< 0.001
No	2584 (76.2)	145 (60.9)	2439 (77.3)	
During first trimester only	318 (9.3)	23 (9.7)	295 (9.4)	
Continued during pregnancy	491 (14.5)	70 (29.4)	421 (13.3)	
Folic acid supplement use, *N* (%)				< 0.001
No	627 (18.5)	89 (37.4)	538 (17.1)	
Start during first 10 weeks of pregnancy	1109 (32.7)	102 (42.9)	1007 (31.9)	
Preconception use	1657 (48.8)	47 (19.7)	1610 (51.0)	
Total daily energy intake, mean (SD), kcal	2076 (535)	2015 (529)	2081 (530)	< 0.01

^a^Values are means (standard deviation) for continuous variables with a normal distribution, or medians (95% range) for continuous variables with a skewed distribution, and valid percentages for categorical variables. Missing values in covariates are imputed. Percentages of pre-pregnancy BMI clinical categories and gestational weight gain clinical categories are valid percentages

^b^*P*-values for differences in subject characteristics between mothers with psychological distress and mothers without psychological distress were calculated performing independent sample *t* tests for normally distributed continuous variables, the Mann-Whitney test for not normally distributed continues variables and chi-square tests for categorical variables

**Table 2:** Psychological distress and gestational weight gain characteristics by pre-pregnancy BMI category (*N* = 3393)^a^

	Pre-pregnancy BMI categories			
	Underweight	Normal weight	Overweight	Obesity
	*N* = 111	*N* = 2127	*N* = 491	*N* = 185
Weight gain measurements				
Total gestational weight gain, mean (SD)	14.4 (5.3)	15.4 (5.3)	14.3 (6.4)	11.5 (8.6)
Weight gain in the second half of pregnancy, mean (SD)	9.1 (4.5)	9.8 (4.3)	9.5 (4.8)	8.0 (5.7)
Psychological measurements				
Psychological distress	9 (8.1%)	140 (6.6%)	40 (8.1%)	15 (8.1%)
No psychological distress	102 (91.9%)	1987 (93.4%)	451 (91.9%)	170 (91.9%)
Depression	10 (9.0%)	134 (6.3%)	41 (8.4%)	10 (5.4%)
No depression	101 (91.0%)	1992 (93.7%)	450 (91.6%)	174 (94.1%)
Anxiety	8 (7.2%)	173 (8.1%)	50 (10.2%)	16 (8.6%)
No anxiety	103 (92.8%)	1954 (91.9%)	441 (89.9%)	169 (91.4%)

Non-response analyses showed that, as compared to women with missing information on weight gain during pregnancy, those with information on gestational weight gain had a higher maternal age, a lower pre-pregnancy BMI, a partner, a higher intake of alcohol and folic acid and a higher total daily energy intake, and were more often nulliparous, higher educated and Dutch-European (Electronic Supplementary Material 2).

### Psychological Distress and Weight Gain in the Second Half of Pregnancy

**Fig. 1:**
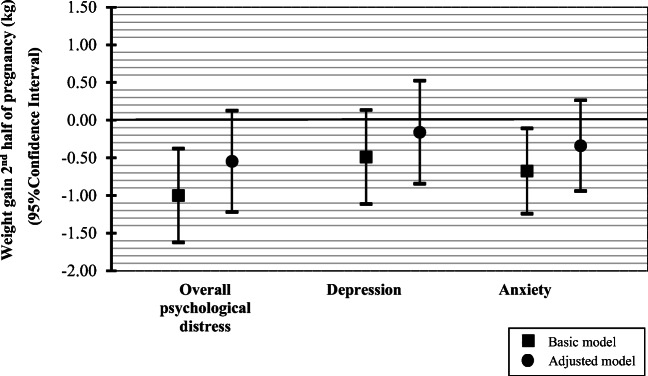
Associations of psychological distress and weight gain in the second half of pregnancy (*N* = 3263). Values are linear regression coefficients (95% confidence intervals) and represent the overall change in weight gain in the second half of pregnancy for psychological distress, depression and anxiety compared to no psychological distress, depression or anxiety. The basic model was adjusted for maternal age. The adjusted model was adjusted for maternal age, pre-pregnancy BMI, parity, education, marital status, ethnicity, alcohol intake, smoking, folic acid use and nutritional intake in kcal

Figure 1 shows that, in the basic models, overall psychological distress and anxiety were associated with lower weight gain in the second half of pregnancy (differences − 1.00 kg (95% confidence interval (CI) − 1.62, − 0.37) and − 0.68 kg (95% CI -1.24, -0.11), for overall psychological distress or anxiety, respectively). These associations fully attenuated into non-significance in the adjusted model after taking account for socio-demographic variables such as maternal education and ethnicity. Maternal depression during pregnancy was not associated with weight gain in the second half of pregnancy.

For the associations of maternal psychological distress, depression and anxiety with total gestational weight gain, effect estimates were of the same magnitude and in similar direction as for weight gain in the second half of pregnancy (Electronic Supplementary Material 3). The stratified analyses for different BMI categories are shown in Electronic Supplementary Material 4. Underweight women experiencing overall psychological distress or depression tended to have an increased weight gain from 20 weeks onwards compared with normal, overweight, and obese women with overall psychological distress or depression; however, results were not significant. In obese women experiencing anxiety during pregnancy, weight gain in the second half of pregnancy tended to be lower. Sensitivity analyses among women who had a full term pregnancy only, showed effect estimates of the same size and direction as the main analyses (Electronic Supplementary Material 5).

### Psychological Distress and the Risk of Inadequate and Excessive Weight Gain in Pregnancy

**Fig. 2:**
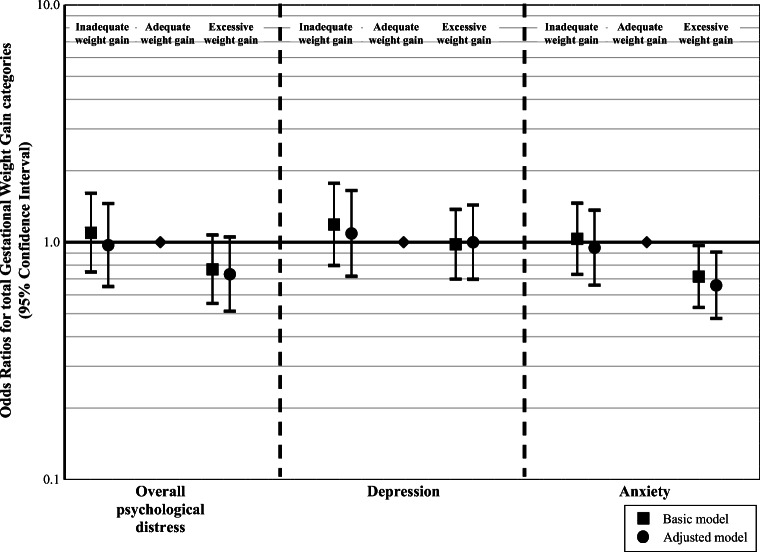
Associations of psychological distress with clinical categories of gestational weight gain (*N* = 2914). Odds ratios (95% confidence intervals) represent the risks for the different weight gain categories (inadequate, adequate (reference) and excessive weight gain) according to the 2009 IOM gestational weight gain recommendation categories for women with overall psychological distress, depression and anxiety. The basic model is adjusted for maternal age. The adjusted model is adjusted for maternal age, pre-pregnancy BMI, parity, educational level, marital status, ethnicity, alcohol intake, smoking, folic acid intake, and nutritional intake in kcal

Overall psychological distress and depression were not associated with the risks of inadequate or excessive weight gain (Fig. 2). Maternal anxiety during pregnancy was, independently of confounders, associated with a lower risk of excessive weight gain (odds ratio 0.61 (95% CI 0.48, 0.91) (Fig. 2). Similar results were obtained without adjustment for pre-pregnancy BMI.

## Discussion

### Main Findings

In this population-based prospective cohort study, we did not observe consistent associations of overall psychological distress, depression, and anxiety with gestational weight gain. Most associations were explained by maternal ethnicity and educational level. Only women with anxiety symptoms had, independently of potential confounders, a lower risk of excessive weight gain.

### Interpretation of Main Findings

We observed that 7.0% to 8.4% of all pregnant women reported psychological distress, depression, or anxiety. These percentages are comparable or slightly lower compared to the prevalence reported in previous studies [2, 3]. In our study population, 23.1% of women had pre-pregnancy overweight or obesity and 20.1% and 45.0% of women experienced inadequate and excessive weight gain, respectively, which is in line with population figures [5, 31–34]. Although results of some previous studies suggested that psychological distress, depression, or anxiety during pregnancy are associated with gestational weight gain [14–17], one systematic review has reported no association [9] and a second systematic review only reported an association of depression, but not psychological distress and anxiety, with gestational weight gain [7]. Most previous studies did not define cutoffs for psychological distress to consider clinical importance and sample sizes were modest. In our large population-based prospective cohort study, we observed that overall psychological distress and anxiety were associated with a lower gestational weight gain. However, these associations attenuated after adjustment for socio-demographic factors. These findings are in line with a study among 1605 women in the USA in which the relationship between psychosocial status and adequacy of gestational weight gain was also influenced by socio-demographic factors [31]. In a large study among 13,314 pregnant women in the UK between 1991 and 1992, the association between antenatal depression and inadequate or excessive gestational weight gain was already non-significant in the unadjusted model [35]. However, this study did not assess the association of overall psychological distress and anxiety with gestational weight gain. One previous study in 242 women found that depression during pregnancy was associated with excessive gestational weight gain, only among women with a high pre-pregnancy BMI [16]. We did not find significant differences in the association of psychological distress, depression, or anxiety with weight gain during pregnancy between women of different pre-pregnancy BMI categories. Thus, results from our and some other studies suggest that common socio-demographic factors explain the association between psychological distress and weight gain during pregnancy.

In the present study, we observed a negative association between anxiety and the risk for excessive weight gain, which remained after adjustment. Our finding is not in line with a recent study among 725 women in which lower reported stress was associated with a greater chance of women achieving adequate gestational weight gain [14]. However, some previous studies also suggest that anxiety and depression may be protective of increased weight gain [15, 17]. In the USA-study mentioned above, the association between anxiety and a higher adequacy of weight gain disappeared after adjustment for confounders, among which was physical activity [31]. In our study, the association between anxiety and the risk for excessive weight gain remained significant. However, residual confounding by for example physical activity level and sedentary behavior may still be present.

The relationship between psychological distress and weight is complex and might be bidirectional [7, 36, 37]. Since observational studies are not able to clarify the causal directions, mechanistic studies and Mendelian Randomization studies may give further insight in the underlying mechanisms and directions [38]. This is of great importance because both psychological health and weight gain can be targets for preventive strategies in pregnancy. This is shown in a randomized controlled trial in which the effects of a four-session intervention, motivating participants to have a healthy lifestyle during pregnancy, were examined [39]. The study found a reduction of gestational weight gain and levels of anxiety in obese pregnant women after the intervention.

### Strengths and Limitations

Strengths of this population-based cohort study were the prospective data collection, the detailed measurements from pregnancy onwards and the large sample size of more than 3000 participants. This study also has limitations. Of all women included during pregnancy, 75% responded to the questionnaire. Only 52% of all women included during pregnancy with information on psychological distress and with singleton live-born children, had information on weight gain during pregnancy. Non-response could have led to selection bias if the associations were different between those included and not included in the analyses. Extrapolating results to all pregnant women should therefore be done with caution. Women with missing information on psychological distress and weight gain were more often lower educated and of non-European ethnicity. We cannot exclude the possibility that these differences have affected the results. Information on gestational weight gain was self-reported. Self-reported weight tends to be underestimated. We used self-reported weights because the measured weights, weight in the first trimester (median 13.2 weeks, 95% range 9.8–17.5 weeks) and weight in the third trimester (median 30.2 weeks, 95% range 28.5–32.6 weeks), do not comprise the whole pregnancy. However, the correlations between self-reported pre-pregnancy weight and measured weight in the first trimester (*r* = 0.96, *P* < 0.001) as well as the correlation between maximum self-reported weight and measured weight in the third trimester were high (*r* = 0.95, *P* < 0.001). Psychological distress was measured at only one time point during pregnancy, on average at 20 weeks, and refers to the preceding 7 days. Most other studies also have only one assessment point [40]. Therefore, we do not know whether psychological distress symptoms varied in intensity or were persistent throughout pregnancy. Further research is needed to assess the associations of trimester-specific psychological distress on gestational weight gain. To classify psychological symptoms, we have used cutoffs derived from a clinical population of Dutch non-pregnant women, which might not be entirely suitable for our study population. However, cutoffs from a sample of pregnant women are currently not available. In this study, differential misclassification could occur when women with more psychological distress, depression or anxiety report differently their weight status compared to women without psychological distress, depression, or anxiety. This seems unlikely because both pregnant women and data collectors were unaware of the specific research questions under study [41]. Finally, although we used a large number of confounders, residual confounding might still be present.

## Conclusions

Our results do not support the hypothesis that psychological distress, depression, and anxiety affect weight gain in pregnant women. Only women with anxiety symptoms had, independently of potential confounders, a lower risk of excessive weight gain. The observed associations of psychological distress with weight gain during pregnancy seem to be largely explained by common socio-demographic factors. Further studies are needed to explore whether psychological distress in pregnancy affects other outcomes in women and their children, such as postpartum weight gain/loss.

## Electronic Supplementary Material

ESM 1 (DOCX 31 kb)

ESM 2 (DOCX 24 kb)

ESM 3 (DOCX 46 kb)

ESM 4 (DOCX 68 kb)

ESM 5 (DOCX 20 kb)

## Abbreviations

BMI: Body mass index
BSI: Brief symptom inventory
IOM: Institute of Medicine
GSI: Global Severity Index
WHO: World Health Organization
