# Supplementary material for: Psychological Distress and Weight Gain in Pregnancy: a Population-Based Study
Source: Int J Behav Med. 2019 Dec 18;27(1):30–8. doi: 10.1007/s12529-019-09832-0 (PMC7058670; doi:10.1007/s12529-019-09832-0)
Supplement: Supplementary file 1 — (DOCX 31 kb) [file 12529_2019_9832_MOESM1_ESM.docx]

**Psychological distress and weight gain in pregnancy:**

**a population-based study**

Florianne O.L. Vehmeijer, MD^1,2^, Sangeeta R. Balkaran, BsC^1,2^, Susana Santos, PhD^1,3^, Romy Gaillard, MD, PhD^1,3^, Janine F. Felix MD, PhD^1,2^, Manon H.J. Hillegers MD, PhD^2,4^, Hanan El Marroun MD,PhD^2,4^, Vincent W.V. Jaddoe MD, PhD^1,3^

1. The Generation R Study Group, Erasmus MC, University Medical Center, Rotterdam, The Netherlands
2. Department of Epidemiology, Erasmus MC, University Medical Center, Rotterdam, The Netherlands
3. Department of Pediatrics, Erasmus MC, University Medical Center, Rotterdam, The Netherlands
4. Department of Child and Adolescent Psychiatry/Psychology, Erasmus MC - University Medical Center, Rotterdam, The Netherlands

Corresponding Author: Vincent W.V. Jaddoe; e-mail: [v.jaddoe@erasmusmc.nl](mailto:v.jaddoe@erasmusmc.nl)

**Electronic Supplementary Material 1. Flowchart of the study participants**

**N *=* 6650**

Women included during pregnancy with information on psychological distress

**N = 101**

Excluded:

Twin pregnancies (N = 73)

Fetal deaths (N = 12)

Lost to follow-up (N = 16)

**N *=* 6549**

Women included during pregnancy with information on psychological distress and with singleton live-born children

**N = 8879**

Women included during pregnancy

**N = 2229**

Excluded: Women without information on overall psychological distress during pregnancy

**N = 3156**

Excluded: Mothers without information on weight gain in pregnancy

**N = 3393**

Women included during pregnancy with information on psychological distress, with singleton live-born children and information on weight gain in pregnancy

Weight gain 2^nd^ half of pregnancy N = 3263

Total weight gain N = 2917
