# Supplementary material for: Psychological Distress and Weight Gain in Pregnancy: a Population-Based Study
Source: Int J Behav Med. 2019 Dec 18;27(1):30–8. doi: 10.1007/s12529-019-09832-0 (PMC7058670; doi:10.1007/s12529-019-09832-0)
Supplement: Supplementary file 2 — (DOCX 24 kb) [file 12529_2019_9832_MOESM2_ESM.docx]

**Psychological distress and weight gain in pregnancy:**

**a population-based study**

Florianne O.L. Vehmeijer, MD^1,2^, Sangeeta R. Balkaran, BsC^1,2^, Susana Santos, PhD^1,3^, Romy Gaillard, MD, PhD^1,3^, Janine F. Felix MD, PhD^1,2^, Manon H.J. Hillegers MD, PhD^2,4^, Hanan El Marroun MD,PhD^2,4^, Vincent W.V. Jaddoe MD, PhD^1,3^

1. The Generation R Study Group, Erasmus MC, University Medical Center, Rotterdam, The Netherlands
2. Department of Epidemiology, Erasmus MC, University Medical Center, Rotterdam, The Netherlands
3. Department of Pediatrics, Erasmus MC, University Medical Center, Rotterdam, The Netherlands
4. Department of Child and Adolescent Psychiatry/Psychology, Erasmus MC - University Medical Center, Rotterdam, The Netherlands

Corresponding Author: Vincent W.V. Jaddoe; e-mail: [v.jaddoe@erasmusmc.nl](mailto:v.jaddoe@erasmusmc.nl)

**Electronic Supplementary Material 2. Characteristics of women with and without information on weight gain in pregnancy (N = 6549)^a^**

|  | **Full group**  **(N=6549)** | **Responders**  **(N=3393)** | **Non-responders**  **(N=3156)** | **P-value^b^** |
| --- | --- | --- | --- | --- |
| Age at intake, mean (SD), years | 30.0 (5.1) | 31.0 (4.7) | 28.9 (5.4) | < 0.001 |
| Missings (%) | 1 (0.02) | 0 (0.0) | 1 (0.03) |  |
| Pre-pregnancy weight, median (95% range), kg | 64.0 (48.0, 99.0) | 64.0 (49.0, 97.0) | 64.0 (48.0, 101.0) | 0.38 |
| Missings (%) | 1023 (15.6) | 476 (14.0) | 547 (17.3) |  |
| Height, mean (SD), cm | 167.6 (7.4) | 168.8 (7.2) | 166.4 (7.3) | < 0.001 |
| Missings (%) | 17 (0.3) | 7 (0.2) | 10 (0.3) |  |
| Pre-pregnancy BMI, median (95% range), kg/m² | 22.6 (18.0, 34.7) | 22.3 (18.2, 33.6) | 22.8 (17.7, 35.7) | <0.001 |
| Missings (%) | 1030 (15.7) | 479 (14.1) | 551 (17.5) |  |
| Pre-pregnancy BMI clinical categories, N (%) |  |  |  | < 0.001 |
| Underweight | 241 (3.7) | 111 (3.3) | 130 (4.1) |  |
| Normal weight | 3788 (57.8) | 2127 (62,7) | 1661 (52.6) |  |
| Overweight | 1018 (15.5) | 491 (14.5) | 527 (16.7) |  |
| Obesity | 472 (7.2) | 185 (5.5) | 287 (9.1) |  |
| Missings | 1030 (15.7) | 479 (14.1) | 551 (17.5) |  |
| Gestational age at birth, median (95% range), weeks | 40.1 (35.9, 42.3) | 40.1 (36.3, 42.4) | 40.1 (35.4, 42.3) | < 0.001 |
| Missings (%) | 2 (0.03) | 0 (0.0) | 2 (0.06) |  |
| Parity,% |  |  |  | < 0.001 |
| Nulliparous | 3786 (57.8) | 2051 (60.4) | 1735 (55.0) |  |
| Multiparous | 2725 (41.6) | 1329 (39.2) | 1396 (44.2) |  |
| Missings, N (%) | 38 (0.6) | 13 (0.4) | 25 (0.8) |  |
| Education, N (%) |  |  |  | < 0.001 |
| Primary school | 576 (8.8) | 166 (4.9) | 410 (13.0) |  |
| Secondary school | 2824 (43.1) | 1237 (36.5) | 1587 (50.3) |  |
| Higher education | 2840 (43.3) | 1914 (56.4) | 926 (29.3) |  |
| Missings, N (%) | 309 (4.7) | 76 (2.2) | 233 (7.4) |  |
| Marital status,% |  |  |  | < 0.001 |
| Married/living together | 5388 (82.3) | 2986 (88.0) | 2402 (76.1) |  |
| No partner | 829 (12.7) | 298 (8.8) | 531 (16.8) |  |
| Missings, N (%) | 332 (5.1) | 109 (3.2) | 223 (7.1) |  |
| Ethnicity, N (%) |  |  |  | < 0.001 |
| Dutch-European | 3954 (60.4) | 2441 (71.9) | 1513 (47.9) |  |
| Surinamese | 550 (8.4) | 197 (5.8) | 353 (11.2) |  |
| Turkish | 513 (7.8) | 174 (5.1) | 339 (10.7) |  |
| Moroccan | 353 (5.4) | 107 (3.2) | 246 (7.8) |  |
| Cape Verdian | 236 (3.6) | 69 (2.0) | 167 (5.3) |  |
| Dutch Antilles | 206 (3.1) | 67 (2.0) | 139 (4.4) |  |
| Others | 592 (9.0) | 317 (9.3) | 275 (8.7) |  |
| Missings, N (%) | 145 (2.2) | 21 (0.6) | 124 (3.9) |  |
| Alcohol consumption, N (%) |  |  |  | < 0.001 |
| No | 2687 (41.0) | 1177 (34.7) | 1510 (47.8) |  |
| Yes | 3222 (49.2) | 1906 (56.2) | 1316 (41.7) |  |
| Missings, N (%) | 640 (9.8) | 310 (9.1) | 330 (10.5) |  |
| Smoking habits, N (%) |  |  |  | < 0.001 |
| No | 4378 (66.8) | 2375 (70.0) | 2003 (63.5) |  |
| During first trimester only | 500 (7.6) | 294 (8.7) | 206 (6.5) |  |
| Continued during pregnancy | 1115 (17.0) | 448 (13.2) | 667 (21.1) |  |
| Missings, N (%) | 556 (8.5) | 276 (8.1) | 280 (8.9) |  |
| Folic acid supplement use, N (%) |  |  |  | < 0.001 |
| No | 1298 (19.8) | 459 (13.5) | 839 (26.6) |  |
| Start during first 10 weeks of pregnancy | 1628 (24.9) | 887 (26.1) | 741 (23.5) |  |
| Preconception use | 2174 (33.2) | 1366 (40.3) | 808 (25.6) |  |
| Missings, N (%) | 1449 (22.1) | 681 (20.1) | 768 (24.3) |  |
| Total daily energy intake, mean (SD), kcal | 2051 (557) | 2094 (525) | 2001 (589) | < 0.001 |
| Missings, N (%) | 1324 (20.2) | 584 (17.2) | 740 (23.4) |  |

**^a^** Values are means (standard deviation) for continuous variables with a normal distribution, or medians (95% range) for continuous variables with a skewed distribution, and valid percentages for categorical variables. Missing values in covariates are imputed.

**^b^** P-values for differences in subject characteristics between responders and non-responders were calculated performing independent sample t-tests for normally distributed continuous variables, Mann-Whitney test for not normally distributed continues variables and chi-square tests for categorical variables.
