# Supplementary material for: Psychological Distress and Weight Gain in Pregnancy: a Population-Based Study
Source: Int J Behav Med. 2019 Dec 18;27(1):30–8. doi: 10.1007/s12529-019-09832-0 (PMC7058670; doi:10.1007/s12529-019-09832-0)
Supplement: Supplementary file 3 — (DOCX 46 kb) [file 12529_2019_9832_MOESM3_ESM.docx]

**Psychological distress and weight gain in pregnancy:**

**a population-based study**

Florianne O.L. Vehmeijer, MD^1,2^, Sangeeta R. Balkaran, BsC^1,2^, Susana Santos, PhD^1,3^, Romy Gaillard, MD, PhD^1,3^, Janine F. Felix MD, PhD^1,2^, Manon H.J. Hillegers MD, PhD^2,4^, Hanan El Marroun MD,PhD^2,4^, Vincent W.V. Jaddoe MD, PhD^1,3^

1. The Generation R Study Group, Erasmus MC, University Medical Center, Rotterdam, The Netherlands
2. Department of Epidemiology, Erasmus MC, University Medical Center, Rotterdam, The Netherlands
3. Department of Pediatrics, Erasmus MC, University Medical Center, Rotterdam, The Netherlands
4. Department of Child and Adolescent Psychiatry/Psychology, Erasmus MC - University Medical Center, Rotterdam, The Netherlands

Corresponding Author: Vincent W.V. Jaddoe; e-mail: [v.jaddoe@erasmusmc.nl](mailto:v.jaddoe@erasmusmc.nl)

**Electronic Supplementary Material 3. Associations of psychological distress with total gestation weight gain (N = 2917)**


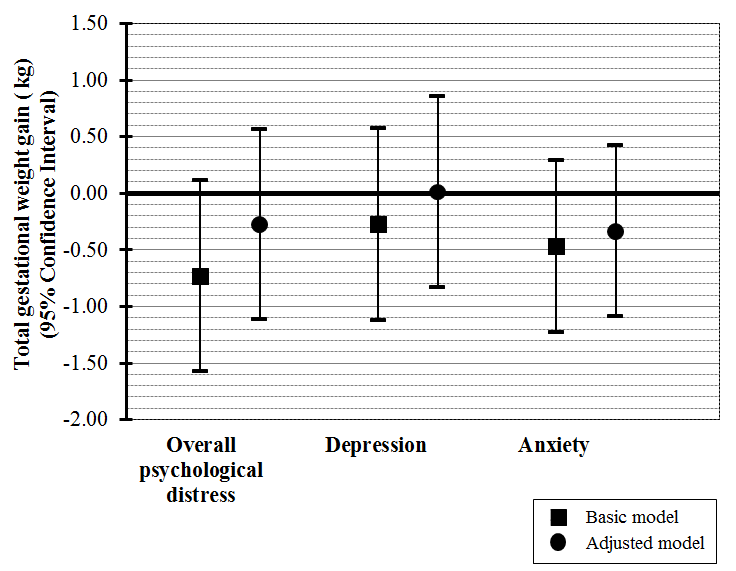


**Online Resource 3.** Values are linear regression coefficients (95% confidence intervals) and represent the overall change in total gestational weight gain for psychological distress, depression and anxiety compared to no psychological distress, depression or anxiety. The basic model was adjusted for maternal age. The adjusted model was adjusted for maternal age, pre-pregnancy BMI, parity, education, marital status, ethnicity, alcohol intake, smoking, folic acid use and nutritional intake.
